# Improving Population Health: The Business Community Imperative

**Published:** 2010-10-15

**Authors:** Andrew Webber, Suzanne Mercure

**Affiliations:** National Business Coalition on Health, Washington, DC; National Business Coalition on Health

## Abstract

Information on the economic effect of poor population health is needed to engage the business community in population health improvement. In a competitive global market, the United States has high health care costs and poor outcomes (measured by such factors as healthy and productive lives) compared with other countries. US business needs to understand population health and not focus just on the health of employees at the worksite. We describe a long-term approach to population health, including incentives, and identify what is needed to engage business leadership in population health improvement.

## The Competitive Challenge

Today, we are spending over $2 trillion a year on health care — almost 50% more per person than the next most costly nation. And yet, as I think many of you are aware, for all of this spending, more of our citizens are uninsured, the quality of our care is often lower, and we aren't any healthier. In fact, citizens in some countries that spend substantially less than we do are actually living longer than we do.

President Barack Obama, Speech to the American Medical Association, June 15, 2009

The US business community competes in a dynamic global economy. The United States has historically achieved success in the global marketplace by excelling at traditional measures of business performance: innovation, technology application, production engineering, capital deployment, marketing, sales, distribution, and customer service. Increasingly, however, 2 related factors put the US business community at a competitive disadvantage: disease burden such as obesity (1) and increases in costs such as health insurance premiums for employers (2).

Business leaders not yet schooled in all the determinants of health (3) and a US health care system biased toward the treatment of illness often say, "With the growing and added investments I am making in health care for my workers and their dependents, surely my company is producing a healthier and more productive workforce." Sadly, this is not the case. As President Obama stated, the United States spends twice as much per citizen on health care as any other country on earth yet ranks in the lowest tier of advanced countries in health outcomes. In other words, the United States produces more health care for less health (4).

A Commonwealth Fund study illustrates more precisely the competitive disadvantage the United States is facing (5). The study demonstrates that the United States, in comparison with other industrialized countries, ranks lowest in metrics of health care that include quality, access, efficiency, and equity indicators; lowest in metrics of long, healthy, and productive lives; and highest in per capita costs. Other data from the *Dartmouth Atlas* (6) show not only wide variation in health care services but that populations in regions with higher spending levels and more physician visits and hospitalizations do not experience better outcomes or quality of care. Seen through this lens, how well the US business community responds to the related challenges of improving health and transforming health care becomes a key driver of market success and of America's future competitiveness and economic security.

This commentary focuses on the role of employers in improving population health. Four issues are addressed: 1) population health from the perspective of employers, 2) incentives for employers to improve population health, 3) opportunities for employers to improve population health, and 4) employers as change agents for improving population health.

## Population Health From the Perspective of Employers

Currently used constructs and measures of population health illustrate the multidimensional nature of the  determinants of population health outcomes. Many of the determinants of health (7,8) are affected, both positively and negatively, by employers, who contribute substantially to population health by generating industrial production, creating jobs and family income, setting employment policies, and influencing health behaviors through worksite cultures, safety practices, and purchasing health care.

Despite their broad influence on population health outcomes, employers' views of population health are narrowly framed by their self-interests. Simply stated, the population that employers care about is their human capital — active employees — followed by employee dependents, and, for the few remaining employers providing generous benefits, their retirees.

Not as central to employers' definition and understanding of population health is community health or the health of the population where employees and their dependents reside. However, business leaders have incentives and compelling reasons to commit to building cultures of health in the worksite and the community. Employers that wish to maximize their influence on human capital as a competitive asset must develop strategies for workforce and community health.

## Incentives for Employers to Improve Population Health

Incentives and rewards are the lifeblood of competitive industries and central to the thinking and culture of business leaders. Moral responsibility and doing the right thing are not dominant factors in corporate decision making. Investment decisions are made by building a business case that an investment today will lead to an economic benefit and a competitive edge tomorrow. The challenge is to broaden the scope of self-interest in building the business case.

Sophisticated employers understand the link between maintenance of workforce health, enhanced productivity, and corporate performance. Building a worksite culture of health with executive leadership, making a sustained commitment to developing human capital, and investing in a spectrum of evidence-based worksite health and health care management programs can increase productivity, reduce employer direct (eg, medical claims) and indirect (eg, absenteeism) costs, and improve bottom-line performance (9). A growing number of business leaders now believe that, in a global economy, workforce health is an important competitive asset that affects employer operating costs and shareholder earnings. For leaders in the nonprofit sector, improving workforce health and productivity is a key driver in advancing any organization's mission.

Incentives to invest in community health are less direct and salient to business leaders than incentives to invest in workforce health. Nevertheless, a compelling business case can and should be made for business leaders to look beyond the worksite to the communities where their organizations do business and their employees reside. Business leaders must understand that an employer can do everything right to influence the health and productivity of its workforce at the worksite, but if that same workforce lives in unhealthy communities, employer investments can be seriously compromised.

Influences on community health and, by extension, workforce health and productivity, include unsafe communities; the presence of a cheap and convenient but a nutritionally unsound food supply; the absence of health education in school curricula and adequate physical education programs; land use and neighborhood design that discourage physical activity and create dependency on car transportation; a health care system with a weak prevention and primary care infrastructure that is oriented toward treatment of acute illness; and poor air and water quality.

Using this broader perspective, the business community's view of population health can radically shift, and strong incentives emerge for employers to invest in community health intervention strategies. What also emerges is an understanding that individual employers do not have the needed leverage on their own to influence community health and health care. Instead, employers must work together collectively and with other community stakeholders on population health strategies that can make a difference. Such an understanding has led during the past several decades to the establishment of business and health coalitions dedicated to improving health and transforming health care, community by community.

The incentives and the business case for employers investing in building healthy communities include the following:

- Improve the health status, and therefore the productivity, of an employer's current and future workforce.
- Control direct (health care) and indirect (absenteeism, disability, presenteeism) costs to the employer.
- Create both the image and the reality of a healthy community that may help recruitment and retention of workforce talent in tight labor markets.
- Increase the buying power and consumption level for business products, in particular nonmedical goods and services, by improving the health and wealth of a community.
- Strengthen an employer's brand and recognition in the community.
- Generate, for individual business leaders, positive feelings of civic pride and responsibility and of being a constructive member of the community.
- Channel corporate philanthropy in a direction that will improve community relations, goodwill, or branding with the potential for a positive return for the business enterprise itself.
- Help create public and private partnerships and a multistakeholder community leadership team that can become the foundation for collaboration, cooperation, and community-based problem solving for many other issues affecting the business community, such as economic development and education.

## Opportunities for Employers to Improve Population Health

**Box. National Business Coalition on Health, Sample of Member Coalitions With Initiatives to Impact Community Population Health**

**Table:** 

**Coalition**	**Coalition-Led Initiative for Community Population Health**
Buyers Health Care Action Group Minneapolis, Minnesota www.bhcag.com	Collaborative initiative with public and private employers to measure and improve health with Healthiest Twin Cities including diagnosis and treatment for chronic conditions and healthier lifestyles
Employers Health Coalition Arkansas Fort Smith, Arkansas www.ehcark.org	Cooperative effort with public health for fluoridation of water to promote oral health
Heartland Healthcare Coalition Morton, Illinois www.hhco.org	Community public campaign to address inappropriate use of antibiotics with employer action component and outreach to primary care physicians.
Louisiana Business Group on Health Baton Rouge, Louisiana www.lbgh.org	Medical home initiative including Medicare and Medicaid to address integrated health care with patient engagement and prevention with emphasis on primary care
Memphis Business Group on Health Memphis, Tennessee www.memphisbusinessgroup.org	Founding member of Healthy Memphis Common Table, which includes consumers, providers, government, and other stakeholders, to address treatment and prevention of obesity and other chronic conditions for a healthier community
Mid-America Coalition on Health Care Kansas City, Missouri www.machc.org	Three-part program to address depression with public education, practitioner engagement for diagnosis and treatment, and worksite initiatives; now leading a Healthier Heartland initiative with multiple stakeholders
Savannah Business Group on Health Savannah, Georgia www.savannahbusiness group.com	Leader in an initiative with city and other stakeholders targeting nutrition, exercise, and obesity with a special focus on schools

Whereas current employer efforts focus on building worksite health promotion initiatives, community-based health improvement strategies are emerging that enjoy the active participation from and leadership of the business community. Many of these initiatives have emerged from employer-based health coalitions that surfaced during the past 3 decades principally to address rising health care costs through *value-based purchasing* (10). Coalitions have learned that community-based organizations collectively representing employers (and their aggregate purchasing power) can provide more leverage on the local health care delivery system than any single company. Now coalitions are applying that same philosophy to influence strategies for broader community health improvement.

Distinct opportunity areas for improving community health quickly surface when employer-led coalitions and members of the National Business Coalition on Health (NBCH) work in partnership with public health officials and other community stakeholders (Box). Many of these partnerships focus on the more clinical aspects of health (eg, cardiovascular health, diabetes, asthma, and depression) but are quickly moving to a more upstream approach focused on primary prevention and better support for healthy lifestyles.

A cross-cutting example is from the Florida Health Care Coalition (FHCC) (11). FHCC, a member of NBCH, partnered with the American Lung Association of Central Florida to bring to the local schools Open Airways for Schools, a school-based asthma risk assessment and health education program for children with asthma in grades 3 through 5 (ages 8-11). FHCC worked with 2 school district members to secure funding for Open Airways instructors to visit the schools and provide asthma education for school officials as well as children. This type of population outreach to dependents of employees — and the broader school community — benefits employers by reducing children's emergency department visits and the associated work time lost by parents. Business-led health coalitions demonstrate creativity and distinctive approaches to improving the health of the population.

## Employers as Change Agents for Improving Population Health

Examples of population health improvement — from workforce to community health improvement — demonstrate that models exist. But what is needed to expand this work, particularly at the community level, and with employers in a leading role? We recommend four distinct needs here: 1) evidence-based interventions, 2) performance incentives, 3) metrics, and 4) business leadership.

### Evidence-based interventions

As business leaders know, success often depends on a good business plan and disciplined execution. As employers become more convinced that they should invest in improving workforce and community health, they will then want to identify the evidence-based intervention strategies that work. Building the evidence base and the lessons learned from a long history of population health strategies and organizing such information so it is easily accessible to community leaders is a priority (12,13).

### Performance incentives

In workforce health improvement initiatives, employers are aggressively implementing incentives to motivate and help move employees and their dependents toward better health. Provider pay-for-performance strategies have become a central and universally recognized element of health care reform legislation and corresponding value-based purchasing initiatives in the private sector. Performance incentives are needed as a catalyst and motivator for community health improvement. With rare exceptions, not enough attention has been paid to strategies and mechanisms that could reward population health improvement (7). Innovative performance incentives should be rapidly explored and tested. Approaches might include making performance-based payments to integrated *accountable care organizations* that can manage population risk or tying the allocation of federal and state public health dollars to communities improving population health status.

### Metrics

Meaningful metrics are an essential ingredient of employer engagement in population health. The field of worksite health has increasingly generated a set of metrics that tie improved workforce health status and reduced illness burden to quantifiable business performance. Similar metrics for community health indicators relevant to business are more elusive.

Typical population health measures relate to length of life, self-reported health status, access to care, disease prevalence, individual health behaviors, socioeconomic factors, and the physical environment. Are these considered meaningful metrics to a business leader? And what is the benefit to business of an improved population health score? Any metric embraced by the employer community needs to speak the language of business. In particular, understanding the revenue benefits of a healthier community is essential, whether the effect comes from reductions in direct health expenditures, improvements in workforce productivity, or customer buying behaviors.

### Leadership

Business leaders go to work each day with this question in mind: "How can I make my company's products and services more competitive in a global economy?" Business leaders do not often think about their company's role as a primary contributor and change agent for improving health and health care. Yet, as key stakeholders with a substantial influence on health and health care, they must — or risk continuation of the status quo. Deteriorating workforce and community health and an expensive and broken health care system affect the bottom line and warrant the immediate attention of business leaders (13). The business community, in its role as employer, health care purchaser, and respected community leader, is in a unique and powerful position to be a change agent. Who else has both the motivation and status in the community to play this key leadership role?

## Conclusion

Poor health and rising health care costs in America are problems in search of employer leadership and solutions. Although many businesses still treat health as an operating cost to be managed, an increasing number of employers — large and small — have begun investing in human capital and building cultures of health at the worksite. There has been less employer attention, leadership, and investment in improving the health of communities and understanding the influence and impact of population health status on business performance. Nevertheless, the work of business and health coalitions indicates that strategies for community health improvement are building momentum and that employers play a lead role. These efforts would be buttressed by more inspired leadership from individual corporate leaders, a stronger evidence base for community health intervention strategies, the establishment of performance incentives for population health, and metrics that speak the language of business.
